# A case report and literature review: pheochromocytoma-mediated takotsubo cardiomyopathy, which is similar to acute myocardial infarction

**DOI:** 10.3389/fcvm.2023.1194814

**Published:** 2023-06-23

**Authors:** Zhiyu Zhang, Xia Guo, Jingyue Wang, Shipeng Wang, Yushi Wang

**Affiliations:** Department of Cardiovascular Medicine, The First Hospital of Jilin University, Changchun, China

**Keywords:** takotsubo cardiomyopathy, AMI, pheochromocytoma, catecholamine, normetanephrine

## Abstract

A 52-year-old Chinese woman was admitted to a cardiac intensive care unit (CCU) due to nausea, vomiting, and dyspnea, which began a day before her hospitalization. Metoprolol succinate and conventional treatment for acute myocardial infarction (AMI) were initially administered to the patient based on electrocardiogram (ECG) findings and elevated cardiac troponin I (cTnI). However, the following day, she developed aggravated nausea, vomiting, fever, sweating, a flushed face, a rapid heart rate, and a significant rise in blood pressure. Furthermore, ultrasonic cardiography (UCG) displayed takotsubo-like changes; nevertheless, ECG indicated inconsistent cTnI peaks with extensive infarction. After coronary computed tomography angiography (CTA) ruled out (AMI), and in conjunction with the uncommon findings, we strongly suspected that the patient had a secondary condition of pheochromocytoma-induced takotsubo cardiomyopathy (Pheo-TCM). In the meanwhile, the use of metoprolol succinate was promptly discontinued. This hypothesis was further supported by the subsequent plasma elevation of multiple catecholamines and contrast-enhanced computed tomography (CECT). After one month of treatment with high-dose Phenoxybenzamine in combination with metoprolol succinate, the patient met the criteria for surgical excision and successfully underwent the procedure. This case report demonstrated that pheochromocytoma could induce TCM and emphasized the significance of distinguishing it from AMI (in the context of beta-blocker usage and anticoagulant management).

## Introduction

Pheochromocytoma is a rare neuroendocrine tumor originating from the chromaffin cells of the adrenal medulla and secretes excessive catecholamines. The catecholamines overproduction can have a direct toxic impact on the myocardium, potentially leading to takotsubo cardiomyopathy (TCM), a condition experienced by approximately 10% of pheochromocytoma patients (1). The distinction between Pheo-TCM and acute myocardial infarction (AMI) could be challenging, especially in patients who had not previously scheduled angiography. Therefore, it is necessary for the clinician to suspect Pheo-TCM timely and responds swiftly according to the patient's symptoms, electrocardiograph (ECG) and myocardial enzyme changes, reaction after medication, and characteristics of cardiac ultrasound. This medical record discussed treating a Pheo-TCM patient and highlighted some experiences.

## Case presentation

A 52-year-old Chinese woman was admitted to (CCU) due to nausea, vomiting, and dyspnea, which began a day before her hospitalization. The patient had no history of smoking, hypertension, diabetes, and coronary heart disease. Moreover, the following were physical examination findings: body temperature: 36.7°C, heart rate: 80 beats/min (bpm), breathing: 18 bpm, and blood pressure: 133/62 mmHg. Auscultation revealed thick breathing sounds in both lungs, and wet rales could be heard in both lungs.

**Figure 1:**
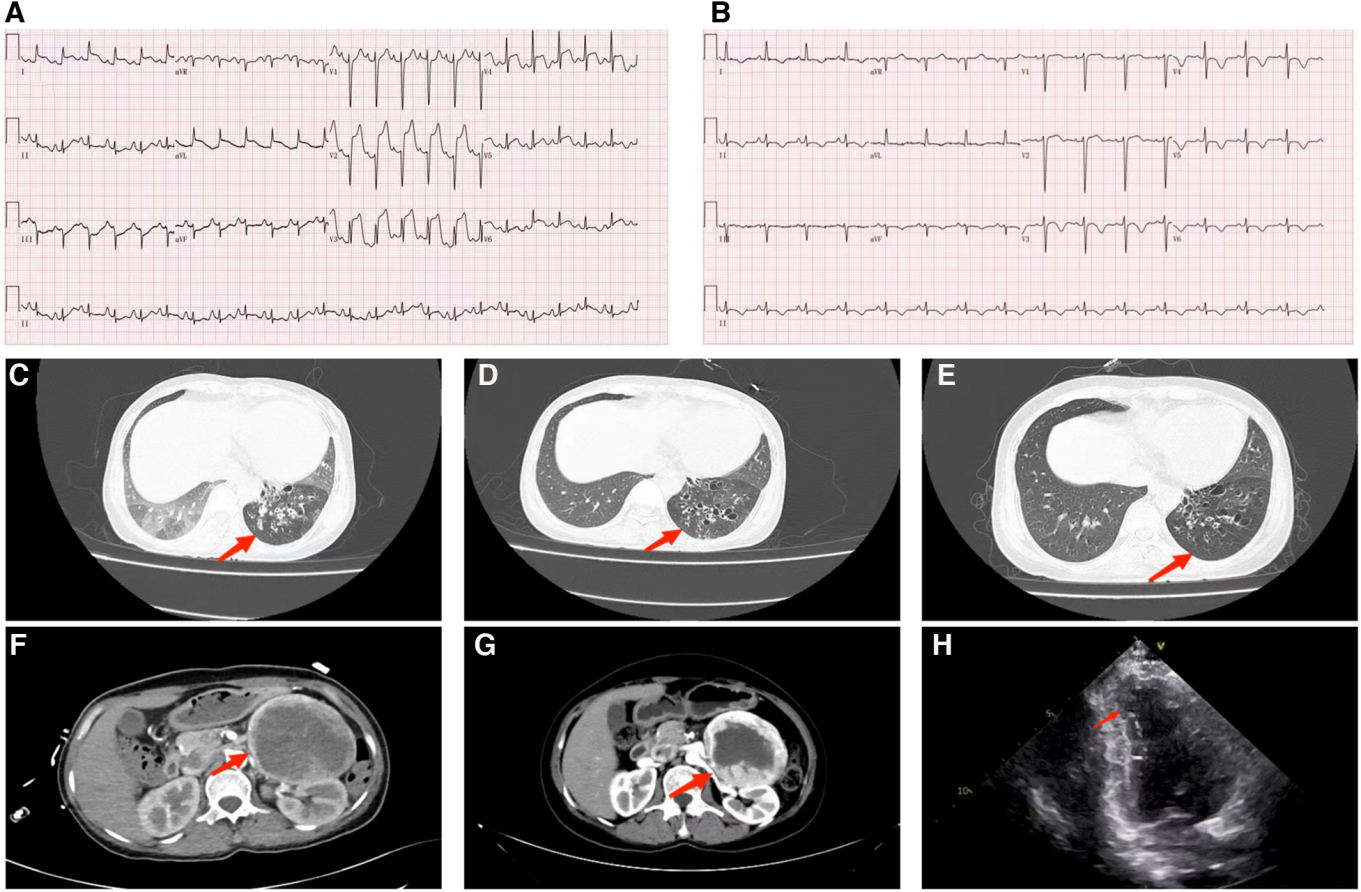
(**A**) ECG of the admitted patient; (**B**) ECG of patients after treatment with phenoxybenzamine. (**C**) Lung CT examination at admission shows that bronchiectasis of the lower lobe of the left lung is associated with infection, bilateral lung inflammation (red arrow). (**D**) Pulmonary CT on the second day after the addition of phenbenamine, displays Bronchiectasis of the lower lobe of the left lung with slight relief of infection (red arrow). (**E**) Preoperative lung CT re-examination depicts that the degree of bronchiectasis with infection in the lower lobe of the left lung is slightly less than that of the previous one (red arrow). (**F**) Abdominal enhanced CT manifests that mass high density mixed density shadow can be observed in the left middle and upper abdomen; the maximum level is about 7.7 cm × 9.4 cm (red arrow). (**G**) Preoperative abdominal CT review, appearing roughly similar to the previous image (red arrow). (**H**) Systolic period in the apical two chamber (A2C) view: displays the apical wall of left ventricle becomes thinner; outward bulg and reduction of amplitude of motion (red arrow).

The initial laboratory data is given in Table 1. Furthermore, the peak level of cardiac troponin I (cTnI) was 27.3 ng/ml. On admission, the ECG (Figure 1) showed elevated ST segments in lead V1–V6. Ultrasonic cardiography (UCG) suggested that the left ventricular end-diastolic (LVED) diameter was 50 mm, ejection fraction (EF) was 52%, and the thickness of the interventricular septum, the middle and lower segment of the interventricular septum, the pulsatile amplitude of the left ventricular (LV) apex and the inferior wall of the LV anterior wall was weakened. The LV apex was slightly dilated outward. Lung computerized tomography (CT) revealed trachea and sputum retention, left lower lung bronchiectasis with infection and bilateral lung inflammation (Figure 1). We considered some inflammatory manifestations of lung CT, which represented pulmonary congestion from acute heart failure.

**Table 1:** The patient's laboratory data.

Parameter	Values	References value	Unit
MYO	390	0–107	ng/ml
TnI	19.7	0–0.05	ng/ml
CKMB	>60	0–4.3	ng/ml
BNP	3,410	0–100	pg/ml
DD	>5,000	100–600	ng/ml
PCT	42.54	0.00–0.500	ng/ml
CRP	68.84	0–1.0	mg/L
pH	7.20	7.35–7.45	–
pCO2	24	35–48	mmHg
pO2	41	83–108	mmHg
Lac	4.1	0.5–2.2	mmol/L
HCO_3_^−^	9.4	18.0–23.0	mmol/L
BE	−16.8	−2.0–3.0	mmol/L
SO2c	63	93–98	%
WBC	19.02	3.50–9.50	10^9^/L
NE	0.93	0.40–0.75	%
NE#	17.66	1.80–6.30	10^9^/L
APTT	27.4	21–33	S
PT	15.3	9.0–13.0	S
INR	1.33	0.8–1.2	–
PTR	1.32	0.8–1.2	–
PTA	60	80–120	%
FBG	4.83	1.8–4.0	g/L
AST	3,308.5	13.0–35.0	U/L
ALT	2,284.7	7.0–40.0	U/L
TP	61.1	65.0–85.0	g/L
ALB	36.9	40.0–55.0	g/L
BUN	20.72	2.6–7.5	mmol/L
sCr	335.5	41–73	umol/L

ALB, albumin; ALT, alanine transaminase; APTT, activated partial thromboplastin time; AST, aspartate aminotransferase; BUN, blood urea nitrogen; BE, base excess; BNP, brain natriuretic peptide; CKMB, creatine kinase myocardial band; CRP, C-reactive protein; DD, D-Dimer; FBG, fibrinogen; INR, international normalized ratio; Lac, lactic acid; MYO, myoglobin; NE, neutrophils; pO2, oxygen partial pressure; pCO2, partial pressure of carbon dioxide; PCT, procalcitonin; pH, potential of hydrogen; PT, prothrombin time; PTR, prothrombin time ratio; PTA, prothrombin activity; SO2c, oxygen saturation; sCr, serum creatinine.TP, total protein; TnI, troponin I; WBC, white blood cell.

The patient was admitted with more than 24 h ST-segment elevation myocardial infarction (STEMI) and pulmonary infection. Considering her stable hemodynamics, absence of chest pain and the strong rejection from his family, we did not immediately perform a coronary angiography on the patient. Furthermore, the patient was given a series of routine treatments, including aspirin (100 mg, QD), clopidogrel (75 mg, QD), enoxaparin sodium (40 mg, Q12 h), metoprolol succinate (23.75 mg, QD), torasemide (20 mg, QD), acetylcysteine (0.6 g, BID), non-invasive ventilator-assisted ventilation and diuresis. Because of concern for infection the patient was treated with piperacillin-tazobactam. The second day following admission, the patient had a fever of 37.6°C, aggravated nausea and vomiting, sudden palpitation, sweating, and a flushing complexion. ECG showed a heart rate of 130 bpm, blood pressure of 207/110 mmHg, and frequent paroxysmal ventricular tachycardia. UCG indicated left atrial enlargement (40 mm × 46 mm × 50 mm), LV enlargement (52 mm), EF: 44%, interventricular septal thickening (12 mm), abnormal LV movement, neck narrowing during LV contraction and balloon-like dilation of the apex of the heart. we immediately improved the coronary artery computed tomography angiography (CTA) examination. The results suggested unobstructed coronary artery flow with no obvious stenosis. Therefore, coronary obstruction as the cause of the AMI was excluded, and TCM was diagnosed based on international takotsubo diagnostic criteria (2). Moreover, low molecular weight heparin and antiplatelet therapy were discontinued. Warfarin was given to prevent apical thrombosis. Since pheochromocytoma could not be ruled out, we replaced metoprolol succinate with amiodarone to control the patient's heart rate and switched from piperacillin-tazobactam to meropenem. Abdominal color ultrasound, plasma catecholamine and its metabolites were detected. Abdominal color ultrasound exposed a heterogeneous echo mass of 94 mm × 90 mm under the left kidney. In addition, an enhanced abdominal CT scan (Figure 1) confirmed the presence of a space-occupying lesion in the posterior peritoneal region of the left mid-epigastric region, which was a neurogenic tumor. In recumbent position, plasma levels of various catecholamines and their metabolites rose significantly, including methoxytyramine 126.9 pg/ml (reference range < 18.4 pg/ml), dopamine 320.6 pg/ml (reference range < 30 pg/ml), norepinephrine 12,377.6 pg/ml (reference range: < 750.0 pg/ml), epinephrine 601.6 pg/ml (reference range: < 111.0 pg/ml), normetanephrine 23,774.9 pg/ml (reference range < 165.0 pg/ml), and metanephrines 12,37.0 pg/ml (reference range < 98.5 pg/ml). The patient was clinically diagnosed with pheochromocytoma and was prescribed phenoxybenzamine (10 mg, QD) and metoprolol succinate (95 mg, QD). To rule out familial pheochromocytoma, the patient's immediate family members were also examined for Plasma fractionated metanephrines and questioned about the relevant clinical manifestations; however, no abnormalities were found.

**Figure 2:**
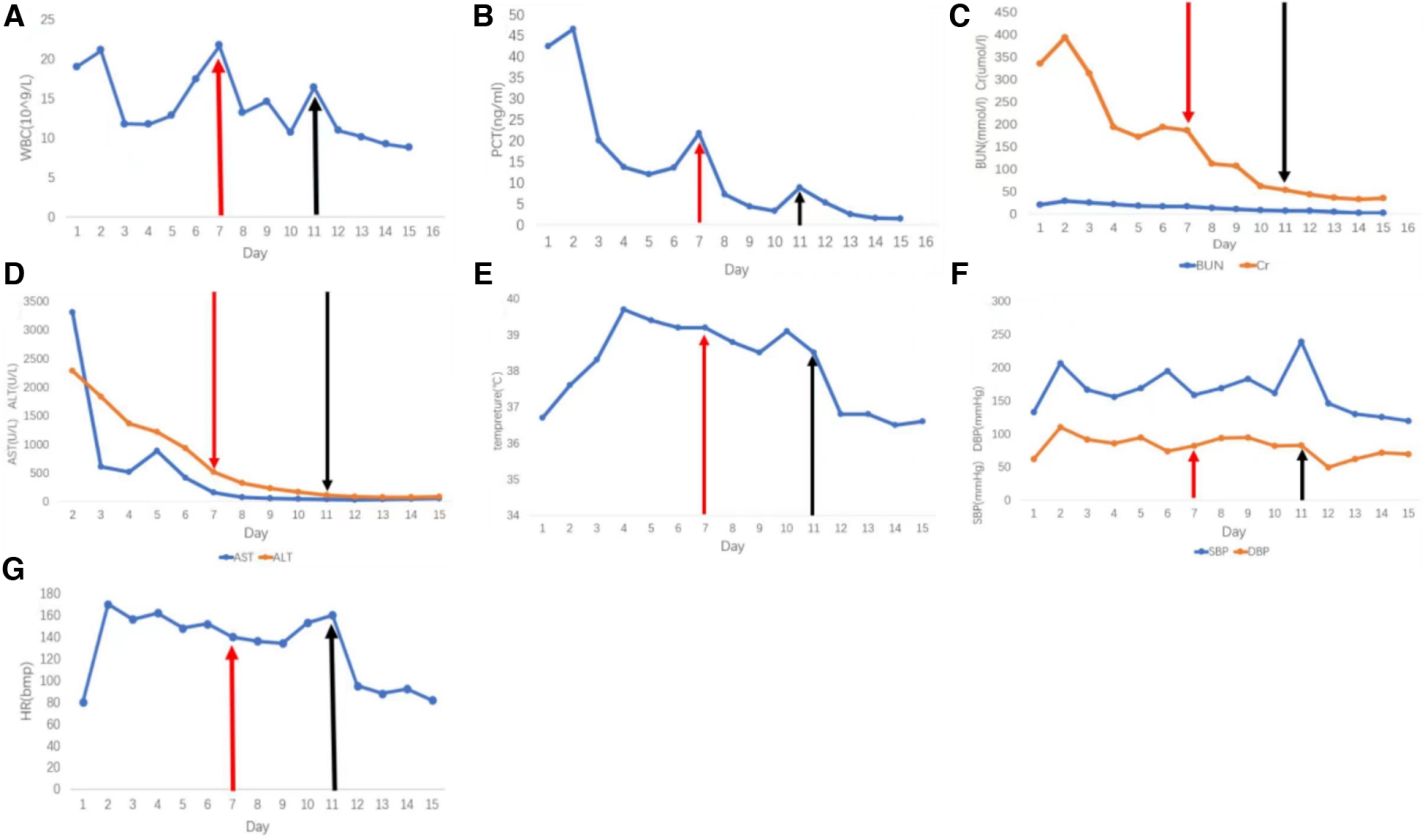
Phenoxybenzamine was used from the 7th day. The red arrow indicates the start of the treatment with phenoxybenzamine, and the black arrow indicates the increase of the dosage of phenoxybenzamine. (**A**) White blood cell count. (**B**) procalcitonin count. (**C**) Blood urea nitrogen and creatinine levels. (**D**) Liver function tests, aspartate transaminase, and alkaline phosphatase. (**E**) Daily maximum temperature. (**F**) Daily maximum systolic and diastolic blood pressure. (**G**) Heart rate.

After adjusting the treatment protocol, the liver and kidney function of the patient and the elevated ST segment of the ECG returned to normal (Figure 1). However, body temperature, blood pressure, and heart rate remained inadequately regulated. To devise an appropriate treatment plan, a whole-hospital discussion was carried out. The Phenoxybenzamine was adjusted to 20 mg three times a day for one month, after which the resection of pheochromocytoma would be evaluated. The primary liver and kidney diseases was excluded. Due to a series of negative pathogenic tests, we suggested further examination of bronchoalveolar lavage fluid to determine the infection's cause. However, the patient's family refused. After 24 h of intensive Phenoxybenzamine therapy, the symptoms of palpitation, sweating, and flushed face were significantly alleviated. Moreover, body temperature, blood pressure and heart rate gradually returned to normal. The lung re-CT examination displayed bilateral lungs had less inflammation than previously (Figure 1); thus, we downgraded meropenem to piperacillin-tazobactam sodium. At the same time, metoprolol succinate was adjusted to 47.5 mg twice a day. As the condition gradually improved, the patient was then discharged from the hospital and instructed to continue oral maintenance treatment with Phenoxybenzamine (20 mg, TID) and metoprolol succinate (47.5 mg, BID). The important laboratory and physical alterations throughout the entire treatment process are detailed in Figure 2.

**Figure 3:**
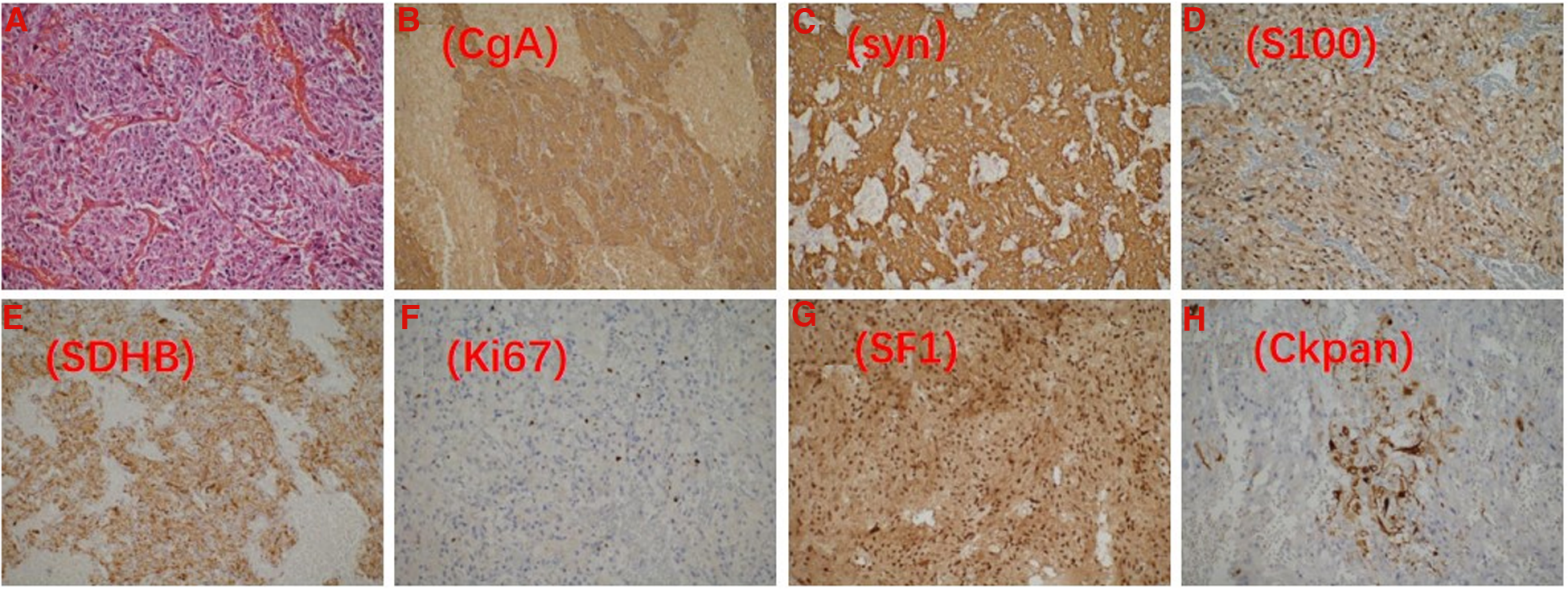
Pathological image. (**A**) HE staining of pheochromocytoma; the tumor is positive for chromogranin-A (**B**) and synaptophysin (**C**); S100 shows a well-developed sustentacular cell network (**D**); The nontumorous elements (e.g. endothelial cells, stromal cells, etc.); SDHB remains positive (**E**); Ki67 (+2%) (**F**). Pheochromocytoma is positive for SF-1 (**G**) and CK-pan (**H**).

After one month, the patient's condition stabilized, and a preoperative evaluation was conducted in our hospital. UCG showed that the LVED was 51 mm, EF was 57%, the thickness of the ventricular septum was 10 mm, and decreased pulsatile amplitude of the basal segment of the LV inferior wall. Other examinations also met surgical requirements (Figure 1). Adrenal pheochromocytoma was confirmed by resection and pathological examination (Figure 3, Pathology). In the outpatient clinic, the UCG were normal one week after the operation.

## Discussion

The American Heart Association (AHA) 2019 scientific statement has excluded TCM from the definition of MINOCA (3). However, TCM is a rare acute heart disease in postmenopausal women, and its clinical features, symptoms, elevated BNP or N-terminal pro-brain natriuretic peptide (NT-proBNP) and ECG are similar to those of AMI (4, 5), thus requiring prompt identification to obtain adequate treatment. Pheochromocytoma is now considered a cause of TCM compared to the diagnostic criteria of the Mayo Clinic (6).

### Pathogenesis

We hypothesized that Pheo-TCM is associated with catecholamine over-release, based on endocardial biopsy evidence of myocardial disturbances and contractile zone necrosis in normal areas of the heart in patients with conditions that increased sympathetic tone (7) and catecholamine-mediated myocardial dysfunction demonstrated in rat models (8). The mechanism may be that rapid increases in plasma catecholamine levels lead to vasospasm and cardiac sympathetic activation, inducing TCM and acute reversible myocardial dysfunction (9). It is also possible that high concentrations of norepinephrine lead to cyclic AMP-mediated calcium overload of the cell, ultimately leading to a decrease in myocardial cell viability (10). In this case, the most significant increase in norepinephrine may be the cause of TCM.

### Clinical characteristics

Chest pain is the most common symptom in Pheo-TCM patients, followed by dyspnoea (11). Some patients also experience symptoms of pheochromocytoma such as headache, palpitation, diaphoresis and hypertension, but all these findings were significantly lower than pheochromocytoma alone (12). The clinical features of Pheo-TCM is not simply a one plus one. Compared to all types of TCM (all-TCM) population (13), Pheo-TCM patients have a younger age (mean age 46.53 ± 15.6 years) (14, 15). Women still dominate, but the proportion is significantly lower (14, 15). The condition is more severe, including a significant decrease in ejection fraction, a significant increase in heart rhythm (14, 15). The complication rate is higher (about three times that of all-TCM)), including heart failure, pulmonary edema, cardiogenic shock (CS), circulatory respiratory failure and apical thrombus formation (14, 15). Due to these complications,the mortality rate of Pheo TCM is high (over 5%) (11). The patient's initial symptoms related to pheochromocytoma were also atypical, indicating that the onset of Pheo-TCM was more insidious.

### Diagnosis

The diagnosis of Pheo-TCM requires a clear diagnosis of pheochromocytoma. Currently, metanephrines, a metabolite of catecholamines, are primarily detected in plasma or urine. Plasma fractionated metanephrines are recommended for patients with a high risk of pheochromocytoma (16). These high-risk patients include patients with resistant hypertension, typical spells, a history of phaeochromocytoma, genetic syndromes, a family history of a genetic syndrome, or an adrenal incidentaloma suggestive of a phaeochromocytoma (16). Plasma fractionated metanephrines are extremely sensitive (17, 18) and pheochromocytoma is excluded if the result is negative, except in preclinical early patients and patients whose tumors secrete only dopamine (17). For patients with low suspicion of pheochromocytoma, 24 h urine fractionated metanephrines is the first-line choice (17). The data available for Pheo-TCM is very limited. Avelyn Aw et al. analyzed 51 Pheo-TCM patients, of which 35 had elevated plasma (nor)metanephrines levels. 16 patients with negative plasma (nor)metanephrines were diagnosed due to elevated urine(nor) metanephrines (*n* = 5) or urine catecholamines (*n* = 11) (11). Patients with positive biochemical results should be further examined by imaging to locate the tumor. CT and magnetic resonance imaging (MRI) of the abdomen and pelvis can be used as initial examination. If the results are negative, complete body radionuclide imaging (such as 18 F-FDOPA PET, [^123^I] metaiodobenzylguanidine (MIBG) scintigraphy, and Ga-68 DOTATATE PET, FDG-PET) should be considered (19). All patients with pheochromocytoma should undergo genetic testing, as they may be sporadic or in the context of genetic syndromes (20). However, our patient declined genetic testing due to financial concerns.

The TCM diagnosis is based on the TCM Consensus Document, coronary angiography with left ventriculography is considered the gold standard (2). Moreover, angiography revealed no evidence of obstructive coronary artery or concomitant coronary artery disease, and the extent of the supply of coronary artery disease did not coincide with the area of abnormal ventricular wall motion, the elevated levels of markers of myocardial damage, such as cTnI and creatine kinase myocardial band (CK-MB) in patients with TCM are significantly lower than would be expected based on the degree of abnormal ventricular wall motion and ECG findings (21). STEMI like changes (36.5%–37.5%) is also the most common ECG changes, but ST-depression (25–26% vs. 8.3%) was higher in Pheo-TCM than All-TCM. The apical or mid-apical pattern was significantly lower (43.9%–45% vs. 83%), but it is still the most common ballooning pattern. Basal (26.2%–30% vs. 2.2%) and gobal (20%–20.6% vs. 0%) pattern was higher in Pheo-TCM than all-TCM. There is no statistically significant difference in mid venturer (14, 15). Cardiac magnetic resonance (CMR) is very useful in the subacute phase of TCM. In addition to identifying regional wall motion abnormalities and distinguishing TCM from other conditions (acute coronary syndrome or many cases of acute myocarditis), CMR can also accurately quantify ventricular function and evaluate the degree of tissue edema, inflammation, necrosis or fibrosis, which is of great help to evaluate the prognosis of patients (22).

In this case, the elevated level of cTnI was inconsistent with the ECG findings of extensive anterior wall myocardial infarction and the patients exhibited neither risk factors for AMI nor chest pain. Following the exclusion of coronary artery disease by CTA, the takotsubo-like changes of the heart color Doppler ultrasound and rapid recovery of ECG and cardiac function after treatment indicated Pheo-TCM. However, the failure to performed timely coronary angiography foreshadowed the aggravation of patients' symptoms the next day and is a lesson worthy of reflection. This suggested that although percutaneous coronary intervention (PCI) does not benefit STEMI patients with stable symptoms for more than 24 h (23), we should not abandon the possibility of using coronary angiography to exclude other similar diseases. In addition, due to the patient's family members, CMR was not employed to obtain a more optimized diagnosis and treatment.

### Treatment and outcomes

Pheo-TCM treatment focuses primarily on etiology and supportive treatment of symptoms. Surgery is the mainstay treatment for Pheo-TCM. AvelynAw et al. found that 98 out of 104 Pheo-TCM patients underwent surgery (11). It is generally accepted that the drug treatment duration for patients with pheochromocytoma prior to surgery is 7–14 days (20). However, for patients with pheochromocytoma crisis (referring to end organ damage including cardiomyopathy, AMI, CS, etc.), through retrospective research and literature analysis, Scholten et al. (24) found that the median time from the onset of pheochromocytoma crisis to surgery was 57 days (range 11–136). They considered that even after an acute event, patients with pheochromocytoma crisis should be stabilized through medication control (adequate alpha-blocker) and be operated within a month instead of emergency surgery (24). Notably, β-blocker drugs alone in patients with pheochromocytoma enhance the vasoconstriction mediated by α1-adrenergic receptors, possibly resulting in a pheochromocytoma crisis (25). As mentioned above, Pheo-TCM condition is more severe, in addition to requiring more positive inotropic drugs (14), some patients may need extracorporeal membrane oxygenation (ECMO) or left ventricular assist device as a bridge to pharmacological therapy and curative adrenalectomy (26, 27). Once the hemodynamics stabilize, these Pheo-TCM patients had a perioperative outcome similar to those with pheochromocytoma without TCM (28). Moreover, due to the thrombo-embolic complications occurred mainly in the apical pattern (14), oral anticoagulants (OAC) should be considered when cTnI > 10 ng/ml in high-risk patients with apical ballooning, although they do not have LV thrombosis (29).

The patient's blood pressure and heart rate increased the day after admission when only beta-blockers were administered. This prompted additional suspicion of pheochromocytoma. Future use of related β-blockers in treating suspected myocardial infarction complicated by pheochromocytoma should be closely monitored. We also concluded that sufficient amount of phenolbenzamine is the key in controlling the patients' symptoms. we also gave the patient diuretics and OAC to reduce heart failure and prevent thrombosis. The drug treatment time was 30 days because the patient was complicated by Severe Pheo-TCM and infection.

## Conclusions

This case demonstrated the importance of identifying Pheo-TCM and AMI, which is related to a series of subsequent patient treatments. Symptoms of patients, hypertension after administration of β-blockers, echocardiographic TCM-like changes, and inconsistencies between myocardial enzymes and ECG helped us to suspect Pheo-TCM. We recommend routine assessment for pheochromocytoma in patients suspected of ACS and not responding to conventionaltherapy.

## Data Availability

The original contributions presented in the study are included in the article, further inquiries can be directed to the corresponding author.
